# Glycosylated Fibronectin in Fetal Growth Restriction: An Emerging Biomarker of Placental Dysfunction

**DOI:** 10.7759/cureus.113553

**Published:** 2026-07-28

**Authors:** Dimitrios I Karoutsos, Konstantinos Stefanidis, Lina Michala, Panagiotis Antsaklis, Despoina Mavrogianni, Georgios Asimakopoulos, Georgios Daskalakis

**Affiliations:** 1 1st Department of Obstetrics and Gynecology, Alexandra Hospital, Athens, GRC

**Keywords:** angiogenic biomarkers, fetal growth restriction, glycosylated fibronectin, placental dysfunction, preeclampsia

## Abstract

Fetal growth restriction (FGR) is a major placenta-mediated pregnancy complication associated with perinatal morbidity, stillbirth, neonatal complications, and long-term cardiometabolic and neurodevelopmental consequences. Its diagnosis remains challenging because fetal smallness alone cannot reliably distinguish constitutionally small fetuses from pathological growth restriction caused by placental insufficiency. Glycosylated fibronectin (GlyFn), a modified extracellular matrix glycoprotein involved in cell adhesion, angiogenesis, endothelial function, and tissue remodeling, has emerged as a promising biomarker of placenta-mediated disease. This review summarizes the biological rationale and current evidence regarding GlyFn in FGR, with emphasis on placental dysfunction, trophoblast invasion, spiral artery remodeling, oxidative stress, endothelial activation, altered angiogenic signaling, and extracellular matrix remodeling. Current data suggest that GlyFn is most consistently associated with preeclampsia, where elevated maternal serum levels have shown diagnostic and prognostic potential. However, direct evidence supporting GlyFn as an independent biomarker of isolated FGR remains limited. Most available findings are derived from preeclampsia cohorts or studies of broader adverse pregnancy outcomes, making it unclear whether GlyFn reflects FGR itself, preeclampsia-related endothelial injury, or general placental stress. GlyFn may be particularly relevant in early-onset placental FGR, where severe malperfusion and endothelial dysfunction are prominent. Its greatest future value may lie in multimarker models combining GlyFn with placental growth factor, soluble fms-like tyrosine kinase-1/placental growth factor ratio, fetal biometry, Doppler indices, and placental histopathology. Prospective longitudinal studies using standardized FGR definitions are required before GlyFn can be translated into clinical practice.

## Introduction and background

Fetal growth restriction (FGR) is a major complication of pregnancy and an important cause of perinatal morbidity and mortality. It describes the failure of a fetus to achieve its expected biological growth potential and should be distinguished from small-for-gestational-age (SGA) status, which is defined primarily according to fetal or neonatal size. Although an estimated fetal weight or abdominal circumference below the 10th percentile is commonly used to identify pregnancies requiring further evaluation, this threshold includes both constitutionally small fetuses and fetuses affected by pathological growth impairment [1,2].

This distinction has important clinical implications. Constitutionally small fetuses may have normal placental function and favorable outcomes, whereas true FGR is associated with impaired placental exchange, fetal hypoxemia, deterioration, and stillbirth. Contemporary diagnostic frameworks therefore combine fetal size with longitudinal growth patterns, maternal risk factors, and Doppler findings suggestive of abnormal placental resistance or fetal circulatory redistribution [2,3]. Nevertheless, no single parameter identifies all affected fetuses, particularly in late-onset disease, in which fetal size may be only moderately reduced and umbilical artery Doppler findings may remain normal.

FGR is commonly classified as early or late onset, according to whether it is diagnosed before or after 32 weeks of gestation [2,4]. Early-onset FGR is less frequent but is generally associated with more severe placental disease, abnormal umbilical artery Doppler indices, hypertensive disorders, and medically indicated preterm delivery. Late-onset FGR is more common and may present with reduced growth velocity, cerebral blood-flow redistribution, or an abnormal cerebroplacental ratio despite relatively preserved umbilical artery Doppler findings. This phenotypic heterogeneity complicates both diagnosis and biomarker evaluation, because a marker associated with severe early placental disease may perform less effectively in milder or later-onset cases.

The clinical consequences of FGR extend beyond reduced fetal size. Growth-restricted fetuses are at an increased risk of intrapartum compromise, stillbirth, preterm delivery, neonatal intensive care admission, respiratory morbidity, hypoglycemia, hypothermia, and impaired neurological development [1,5]. FGR has also been associated with increased susceptibility to hypertension, insulin resistance, cardiovascular disease, and other metabolic disorders later in life. Timely identification is therefore essential for determining surveillance intensity, optimizing the timing of delivery, and reducing preventable adverse outcomes.

Placental insufficiency underlies many cases of pathological FGR. Inadequate extravillous trophoblast invasion and incomplete transformation of the spiral arteries may produce a high-resistance uteroplacental circulation and unstable intervillous perfusion [6]. The resulting oxidative stress, inflammatory signaling, syncytiotrophoblast injury, and angiogenic imbalance may impair maternal-fetal exchange and contribute to both fetal compromise and maternal endothelial dysfunction. These abnormalities are often most pronounced in early-onset FGR and in pregnancies complicated by coexisting preeclampsia.

The assessment of suspected FGR currently relies mainly on ultrasound-based methods. Serial fetal biometry is used to evaluate growth trajectory, while amniotic fluid assessment and Doppler velocimetry provide information on placental resistance and fetal adaptation [1,2]. Uterine and umbilical artery Doppler can identify abnormal uteroplacental or fetoplacental circulation, whereas evaluation of the middle cerebral artery, cerebroplacental ratio, and ductus venosus may help characterize redistribution and hemodynamic compromise. However, these measurements do not directly quantify the molecular processes occurring within the placenta, and their performance may vary according to gestational age, phenotype, measurement variability, and timing of assessment.

These limitations have encouraged the investigation of maternal circulating biomarkers. Placental growth factor (PlGF) and soluble fms-like tyrosine kinase-1 (sFlt-1) are the most extensively evaluated angiogenic markers in placenta-mediated disease [7]. Low PlGF concentrations and an elevated sFlt-1/PlGF ratio may support the diagnosis or prediction of preeclampsia and identify pregnancies at increased risk of adverse outcomes. However, angiogenic imbalance represents only one component of placental dysfunction. Biomarkers reflecting extracellular matrix (ECM) remodeling, trophoblast injury, inflammation, or endothelial activation may provide complementary information and potentially improve discrimination between constitutional smallness and pathological FGR.

Fibronectin is a large ECM glycoprotein involved in cell adhesion, migration, differentiation, tissue repair, angiogenesis, and matrix organization. It exists in several molecular forms generated through alternative splicing and post-translational modification. Changes in fibronectin expression, distribution, or glycosylation may occur in response to tissue injury, inflammation, vascular dysfunction, and altered matrix turnover, providing a biological rationale for investigating modified fibronectin isoforms as circulating markers of placental and endothelial pathology.

Glycosylated fibronectin (GlyFn) has attracted particular interest in obstetric research. Maternal GlyFn concentrations have been evaluated primarily in women with suspected or established preeclampsia, and several studies have reported associations with clinical disease and adverse pregnancy outcomes [8-12]. Interpretation in relation to fetal growth, however, remains complex. Preeclampsia and FGR frequently coexist, especially in early and severe placental disease, but they are distinct clinical entities. Elevated GlyFn in pregnancies complicated by both conditions may reflect maternal endothelial dysfunction, placental ECM remodeling, trophoblast injury, or a combination of these mechanisms.

Evidence specifically addressing GlyFn in isolated FGR remains limited. In many available cohorts, FGR, SGA birth, low birth weight, indicated preterm delivery, and other neonatal outcomes are incorporated into composite endpoints. Differences in diagnostic criteria, gestational age at sampling, disease severity, coexisting hypertension, and outcome definitions further limit comparisons across studies [8-11]. It is therefore essential to separate evidence derived directly from isolated FGR from findings extrapolated from preeclampsia or heterogeneous high-risk pregnancy populations.

The present review critically examines the potential relevance of GlyFn to FGR. It considers the biological relationship between fibronectin glycosylation and placental pathology, evaluates the available clinical evidence, and assesses whether GlyFn may provide information beyond established angiogenic, ultrasound, and Doppler markers. Particular attention is given to the distinction among isolated FGR, preeclampsia-associated FGR, constitutionally small fetuses, and cases supported by abnormal Doppler findings or placental histopathology.

Methods

This article was prepared as a narrative review of the current literature on GlyFn in FGR and related placenta-mediated pregnancy complications. This is a narrative review and was not conducted according to the Preferred Reporting Items for Systematic Reviews and Meta-Analyses (PRISMA) guidelines for systematic reviews. The review aimed to synthesize the available biological, clinical, and translational evidence rather than to perform a formal systematic review or meta-analysis.

Relevant publications were identified through targeted searches of PubMed/MEDLINE, Scopus, and Web of Science. The final literature search was conducted on July 15, 2026. Reference lists of key original studies, systematic reviews, meta-analyses, and clinical guidelines were also screened to identify additional relevant publications.

The principal PubMed/MEDLINE search strategy was:

(“glycosylated fibronectin” OR “fibronectin”) AND (“fetal growth restriction” OR “intrauterine growth restriction” OR “small for gestational age” OR “placental dysfunction” OR “placental insufficiency” OR “preeclampsia” OR “pre-eclampsia” OR “angiogenic biomarkers” OR “placental growth factor” OR “PlGF” OR “soluble fms-like tyrosine kinase-1” OR “sFlt-1” OR “sFlt-1/PlGF ratio”).

The search strategy was adapted as necessary for Scopus and Web of Science. Additional focused searches combined “glycosylated fibronectin” or “fibronectin” with terms including “extracellular matrix remodeling,” “trophoblast dysfunction,” “endothelial activation,” “oxidative stress,” “placental pathology,” “Doppler,” and “adverse pregnancy outcomes.”

Eligible publications included peer-reviewed original clinical studies, mechanistic or translational studies, systematic reviews, meta-analyses, and relevant clinical guidelines addressing GlyFn, fibronectin biology, FGR, SGA, preeclampsia, placental insufficiency, angiogenic imbalance, ECM remodeling, endothelial dysfunction, or related placenta-mediated pregnancy complications. Studies were considered when they provided information on the biological basis, diagnostic performance, prognostic value, clinical applicability, or methodological limitations of GlyFn or related biomarkers.

Because direct evidence concerning GlyFn in isolated FGR remains limited, studies involving preeclampsia, hypertensive disorders of pregnancy, and other high-risk or placenta-mediated pregnancy populations were also included when they provided biologically or clinically relevant indirect evidence. Evidence directly addressing isolated FGR was distinguished from findings derived from preeclampsia-centered cohorts, mixed high-risk populations, or composite obstetric outcomes.

Publications were excluded if they were unrelated to human pregnancy, did not address fibronectin, GlyFn, FGR, placental dysfunction, or related biological pathways, provided insufficient information for interpretation, or represented duplicate reports of the same study population without additional relevant findings. Conference abstracts, editorials, commentaries, letters without original data, case reports, and non-peer-reviewed publications were generally excluded. Only publications written in English were considered. No formal restriction based on publication year was applied.

The included literature was narratively synthesized according to thematic relevance. Particular emphasis was placed on the biological plausibility of GlyFn as a marker of placental and endothelial dysfunction, its relationship with established angiogenic, ultrasound, and Doppler markers, and the evidence gaps limiting its clinical translation. Differences among isolated FGR, preeclampsia-associated FGR, constitutionally small fetuses, and heterogeneous placenta-mediated pregnancy complications were considered throughout the synthesis.

As a narrative review, no formal quality assessment or risk-of-bias evaluation of the included studies was performed, and the synthesis is subject to author interpretation. In addition, the absence of a predefined systematic protocol, duplicate study selection, standardized data extraction, and quantitative evidence synthesis may have introduced selection bias. The findings should therefore be interpreted as a critical narrative synthesis rather than as a systematic review or meta-analysis.

**Figure 1:**
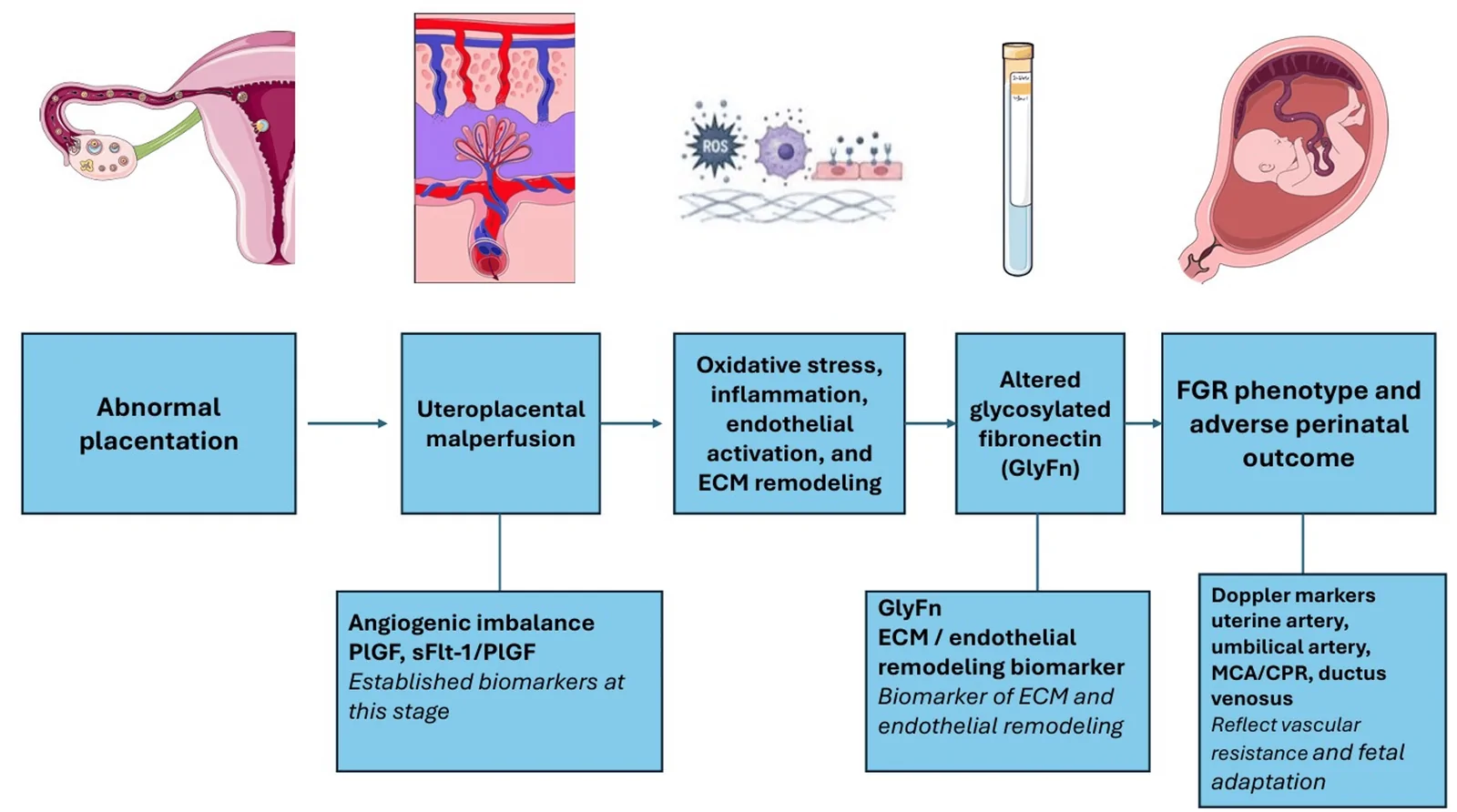
Conceptual model of glycosylated fibronectin in fetal growth restriction. Abnormal placentation may lead to uteroplacental malperfusion, oxidative stress, inflammation, endothelial activation, and extracellular matrix remodeling. These processes may contribute to altered circulating glycosylated fibronectin levels, reflecting placental and endothelial dysfunction. Glycosylated fibronectin may complement established angiogenic biomarkers and Doppler markers in the assessment of placenta-mediated fetal growth restriction. ECM, extracellular matrix; FGR, fetal growth restriction; GlyFn, glycosylated fibronectin; MCA, middle cerebral artery; CPR, cerebroplacental ratio; PlGF, placental growth factor; ROS, reactive oxygen species; sFlt-1, soluble fms-like tyrosine kinase-1. Parts of the figure were generated by using Servier Medical Art (https://smart.servier.com), licensed under CC BY 4.0 (https://creativecommons.org/licenses/by/4.0/) [13].

Figure 1 summarizes the proposed biological framework linking abnormal placentation, uteroplacental malperfusion, endothelial activation, ECM remodeling, altered glycosylated fibronectin, and the fetal growth restriction phenotype.

## Review

Fetal growth restriction: definition and clinical relevance

FGR is a pathological condition in which a fetus fails to achieve its biologically determined growth potential. The term has largely replaced “intrauterine growth restriction” because it more accurately encompasses growth impairment arising from maternal, placental, fetal, or environmental factors. Importantly, reduced fetal size alone does not necessarily indicate disease: some fetuses are constitutionally small but healthy, whereas others are small because of placental insufficiency, chronic hypoxemia, impaired nutrient transfer, or fetal pathology [1-3]. Contemporary guidelines therefore define FGR as a clinical syndrome associated with impaired growth potential and an increased risk of adverse outcomes rather than solely as a biometric abnormality.

An estimated fetal weight (EFW) or abdominal circumference (AC) below the 10th percentile for gestational age is commonly used to identify suspected FGR [1,2]. The Society for Maternal-Fetal Medicine defines FGR as an EFW or AC below the 10th percentile, whereas small-for-gestational-age (SGA) generally refers to a neonate with a birth weight below the same threshold [1]. However, percentile-based definitions may incorrectly classify constitutionally small fetuses as growth restricted and may fail to identify fetuses whose measurements remain above the 10th percentile despite a substantial decline in growth velocity. Modern diagnostic frameworks therefore incorporate fetal size, longitudinal growth, Doppler abnormalities, and evidence of placental dysfunction [2,3].

The distinction between SGA and pathological FGR has direct clinical implications. SGA indicates small size but does not establish placental disease, whereas FGR implies failure to reach individual growth potential and is associated with hypoxemia, acidosis, stillbirth, neonatal morbidity, and long-term developmental consequences [2,3]. Constitutionally small fetuses may require observation without intensive intervention, whereas pathological FGR may necessitate close Doppler surveillance, antenatal corticosteroids when preterm delivery is anticipated, and carefully timed delivery [1,2].

An international Delphi consensus proposed separate diagnostic criteria for early- and late-onset FGR [3]. Early-onset FGR, diagnosed before 32 weeks, may be defined by an AC or EFW below the third percentile or by fetal size below the 10th percentile combined with abnormal uterine or umbilical artery Doppler findings. Late-onset FGR, diagnosed at or after 32 weeks, may be identified through severe fetal smallness or a combination of reduced size, crossing of growth centiles, and evidence of fetal circulatory redistribution, including a reduced cerebroplacental ratio or increased umbilical artery pulsatility index [3].

Early- and late-onset FGR differ in severity, presentation, and surveillance requirements. Early-onset disease is less common but is typically associated with severe placental dysfunction, maternal vascular malperfusion, hypertensive disorders, abnormal uterine and umbilical artery Doppler findings, and preterm delivery [2,4]. Management often requires the integration of fetal Doppler findings, computerized cardiotocography where available, ductus venosus assessment, maternal condition, and gestational age [2]. Late-onset FGR is more common but may be more difficult to recognize because umbilical artery Doppler findings often remain normal. Evidence of fetal adaptation may instead include cerebral vasodilation and a reduced cerebroplacental ratio [2,4].

FGR is associated with chronic hypoxemia, intrapartum compromise, emergency cesarean delivery, neonatal acidosis, hypoglycemia, thermoregulatory instability, respiratory morbidity, feeding difficulties, and admission to the neonatal intensive care unit [1,2,5]. The risk is particularly high in fetuses with severe smallness, abnormal Doppler findings, oligohydramnios, or maternal hypertensive disease [1,2]. Prenatal detection is also central to stillbirth prevention because undiagnosed FGR is disproportionately represented among stillbirths [2].

The consequences may extend into childhood and adulthood. According to the developmental origins of health and disease hypothesis, adverse intrauterine conditions may influence organ development, vascular function, endocrine regulation, and metabolic programming [5]. Individuals affected by FGR have an increased susceptibility to neurodevelopmental impairment, insulin resistance, obesity, hypertension, coronary artery disease, and other cardiometabolic disorders [5].

Accurate definition and classification are therefore essential in both clinical practice and biomarker research. Definitions based exclusively on fetal size may include constitutionally small fetuses without placental disease, thereby weakening associations between candidate biomarkers and true placental dysfunction. Criteria incorporating growth velocity, Doppler abnormalities, and adverse perinatal outcomes may more accurately identify the FGR phenotypes in which biomarkers of endothelial activation, ECM remodeling, and placental pathology are biologically relevant.

Pathophysiology of fetal growth restriction

The pathophysiology of FGR is heterogeneous, but placental dysfunction is central to many clinically significant cases. Placenta-mediated FGR involves abnormal placental development, impaired uteroplacental perfusion, oxidative and inflammatory stress, altered angiogenic signaling, and fetal adaptation to restricted oxygen and nutrient availability. Although maternal, fetal, genetic, infectious, and environmental factors may contribute to FGR in general, defective placentation is particularly important in early-onset and severe FGR [6,14,15].

Normal placentation requires coordinated trophoblast proliferation, differentiation, invasion, and vascular transformation. Extravillous trophoblasts normally replace the endothelial and smooth muscle components of the maternal spiral arteries, converting them into dilated, low-resistance vessels. In placenta-mediated FGR, incomplete trophoblast invasion and spiral artery remodeling result in high-resistance uteroplacental circulation, intermittent perfusion, and reduced placental exchange [6,15].

Impaired trophoblast invasion may be influenced by genetic susceptibility, altered maternal immune tolerance, dysregulated decidual natural killer cell activity, abnormal cytokine signaling, and disturbances in local oxygen tension [6,16]. The resulting placental pathology may include distal villous hypoplasia, accelerated villous maturation, infarction, increased syncytial knotting, decidual arteriopathy, and intervillous fibrin deposition [15,17]. These abnormalities fall predominantly within the standardized category of maternal vascular malperfusion established by the Amsterdam Placental Workshop Group [17].

Uteroplacental malperfusion exposes the placenta to unstable oxygen delivery and ischemia-reperfusion injury. This can damage the syncytiotrophoblast, reduce villous branching and exchange surface area, and impair the transport of oxygen, glucose, amino acids, and other nutrients [6,13]. Clinically, these changes may be associated with abnormal uterine artery Doppler findings, increased umbilical artery resistance, oligohydramnios, and progressive fetal circulatory adaptation.

Oxidative stress is a major consequence of placental malperfusion. Excessive reactive oxygen species production can overwhelm antioxidant defenses and cause lipid peroxidation, protein oxidation, mitochondrial dysfunction, DNA damage, and endoplasmic reticulum stress [6,18]. These processes may suppress placental protein synthesis, impair trophoblast turnover, and reduce placental growth and functional capacity.

Placental stress also promotes the release of inflammatory mediators, syncytiotrophoblast-derived extracellular vesicles, oxidized molecules, and antiangiogenic factors into the maternal circulation. These factors may activate maternal endothelial cells, reduce nitric oxide bioavailability, increase vascular tone, and promote systemic vascular dysfunction [18,19]. Although endothelial dysfunction is most prominent in preeclampsia, related mechanisms are relevant to FGR because both disorders frequently arise from abnormal placentation and maternal vascular malperfusion [6,19].

Inflammatory pathways may further impair trophoblast function, vascular remodeling, and ECM homeostasis. Oxidative injury and syncytiotrophoblast damage stimulate the release of cytokines, chemokines, damage-associated molecular patterns, and cellular debris, thereby amplifying local and systemic inflammatory and endothelial responses [18,19].

Angiogenic imbalance is another major mechanism of placenta-mediated disease. Normal placental vascular development depends on the equilibrium between proangiogenic factors, including vascular endothelial growth factor and placental growth factor (PlGF), and antiangiogenic factors, particularly soluble fms-like tyrosine kinase-1 (sFlt-1). Increased sFlt-1 binds and neutralizes vascular endothelial growth factor and PlGF, reducing angiogenic signaling and contributing to endothelial dysfunction [20]. Although this mechanism is best established in preeclampsia, it is also relevant to early-onset FGR associated with severe placental vascular disease [7,20].

The angiogenic profile varies across FGR phenotypes. Early-onset FGR is more frequently associated with reduced PlGF, elevated sFlt-1, and an increased sFlt-1/PlGF ratio, whereas late-onset disease may exhibit subtler abnormalities [2,4]. This heterogeneity indicates that no single biomarker is likely to capture the full biological spectrum of FGR. Markers reflecting angiogenic imbalance, inflammation, oxidative stress, endothelial activation, and ECM remodeling may therefore provide complementary information.

ECM remodeling is directly relevant to the investigation of glycosylated fibronectin. Fibronectin regulates cell adhesion, migration, angiogenesis, wound repair, trophoblast invasion, decidual remodeling, vascular transformation, and villous development. Placental malperfusion, trophoblast injury, inflammation, and endothelial activation may alter matrix turnover and fibronectin expression. Glycosylated fibronectin may therefore represent a circulating marker of placental-endothelial stress, although direct evidence supporting its role in isolated FGR remains limited.

The fetal response to placental insufficiency is initially compensatory. As oxygen and nutrient availability decline, cardiac output is redistributed toward the brain, myocardium, and adrenal glands. This “brain-sparing” response may be reflected by a reduced middle cerebral artery pulsatility index or cerebroplacental ratio. With progressive deterioration, compensatory mechanisms may fail, leading to abnormal ductus venosus Doppler findings, impaired cardiac function, acidemia, and an increased risk of stillbirth [2,4].

Fibronectin and glycosylated fibronectin: biological background

Fibronectin is a large, multifunctional ECM glycoprotein with central roles in cell adhesion, migration, differentiation, angiogenesis, wound repair, and tissue remodeling. It exists mainly in two forms: soluble plasma fibronectin, which is produced predominantly by hepatocytes and circulates in the blood, and cellular fibronectin, which is produced by several cell types, including fibroblasts, endothelial cells, smooth muscle cells, decidual cells, and trophoblast-related tissues. Structurally, fibronectin is a dimeric glycoprotein composed of two similar polypeptide chains joined by disulfide bonds. It contains multiple binding domains that interact with integrins, collagen, fibrin, heparin, proteoglycans, and other ECM molecules, allowing it to function as a molecular scaffold that coordinates cell-matrix communication [21,22]. Fibronectin is therefore not merely a passive structural component of the ECM, but an active regulator of cellular behavior and tissue organization.

One of the most important biological functions of fibronectin is the mediation of cell adhesion through integrin binding. The arginine-glycine-aspartate motif within fibronectin is recognized by several integrins, particularly α5β1 and αvβ3, which link the ECM to the intracellular cytoskeleton. Through these interactions, fibronectin influences cell spreading, migration, survival, proliferation, and mechanotransduction [21,23]. Integrin-mediated fibronectin signaling is especially relevant in tissues undergoing active remodeling, including the placenta, where trophoblast invasion, decidual transformation, and vascular remodeling require tightly regulated cell-matrix interactions. Disturbances in these interactions may impair trophoblast migration and invasion, contributing to abnormal placentation and placenta-mediated pregnancy complications [24,25].

Fibronectin also plays a fundamental role in ECM assembly and stability. Cellular fibronectin is secreted as a soluble dimer and assembled into insoluble fibrillar networks through a cell-mediated process involving integrins, cytoskeletal tension, and conformational changes in the fibronectin molecule [21,22]. This fibrillar matrix provides a framework for the deposition of other ECM proteins, including collagen and proteoglycans, and contributes to the mechanical properties of tissues. Because fibronectin conformation is sensitive to mechanical stress and molecular binding partners, it may act as a dynamic sensor of tissue remodeling and injury [21]. In the placenta, where the ECM undergoes continuous remodeling throughout gestation, altered fibronectin expression, organization, or molecular modification may reflect maladaptive placental development, abnormal vascular transformation, or tissue stress.

The role of fibronectin in angiogenesis is particularly relevant to placental development and fetal growth. Angiogenesis requires endothelial cell adhesion, migration, proliferation, lumen formation, and stabilization of newly formed vessels, all of which are influenced by ECM composition and integrin signaling. Fibronectin and its receptors are essential components of the angiogenic microenvironment and regulate endothelial cell behavior during developmental and pathological vessel formation [23,26]. Experimental data indicate that fibronectin can support endothelial migration through β1-integrin-dependent pathways and can interact with growth factor signaling systems, including fibroblast growth factor receptor pathways [27]. In the developing placenta, adequate vascular growth and branching are essential for efficient maternal-fetal exchange. Therefore, dysregulated fibronectin-mediated angiogenesis may contribute to impaired placental vascular development and reduced transport capacity in placenta-mediated FGR.

Fibronectin is also closely linked to endothelial function. Endothelial cells are anchored to the vascular basement membrane and surrounding ECM through integrin-mediated adhesions, and these interactions influence vascular barrier integrity, endothelial activation, inflammation, and responses to shear stress. Fibronectin-rich matrices may promote a more activated endothelial phenotype compared with basement membrane components such as laminin or collagen IV [23,28]. This has potential relevance in pregnancy complications characterized by endothelial dysfunction, including preeclampsia and severe placenta-mediated FGR. In these conditions, placental stress may lead to maternal systemic endothelial activation, altered vascular tone, increased vascular permeability, and enhanced inflammatory signaling. Fibronectin, as both an ECM component and a circulating glycoprotein, may therefore reflect vascular injury and remodeling at the maternal-placental interface.

During pregnancy, fibronectin participates in several key reproductive processes, including implantation, trophoblast invasion, decidualization, placental villous development, and vascular remodeling. The ECM at the maternal-fetal interface provides biochemical and mechanical cues that regulate trophoblast differentiation and invasion. Fibronectin supports trophoblast adhesion and migration through integrin-dependent mechanisms [24,25]. However, its biological effects appear context-dependent. Appropriate fibronectin expression and matrix organization are necessary for normal placentation, whereas excessive, deficient, or abnormally modified fibronectin may disrupt trophoblast behavior and vascular adaptation. Pregnancy-focused ECM reviews emphasize that abnormal ECM composition and remodeling are implicated in adverse outcomes, including preeclampsia and fetal growth restriction [24,25].

Glycosylated fibronectin (GlyFn) refers to fibronectin molecules carrying specific carbohydrate modifications. Glycosylation is one of the most common post-translational modifications of proteins and can profoundly influence protein folding, stability, solubility, receptor binding, degradation, immune recognition, and biological activity. Importantly, glycosylation should be distinguished from glycation. Glycosylation is an enzymatically regulated modification that attaches defined carbohydrate structures to proteins, whereas glycation is a non-enzymatic reaction between reducing sugars and proteins. In the case of fibronectin, altered glycosylation may modify its interactions with integrins, ECM proteins, growth factors, and lectin-like receptors. Thus, GlyFn may represent not only the abundance of fibronectin but also qualitative changes in its molecular structure and biological behavior. This distinction is particularly important in pregnancy, where glycosylation patterns may shift in response to placental development, inflammation, hypoxia, oxidative stress, and endothelial activation [9,29].

From a biological perspective, the relevance of GlyFn to placenta-mediated complications may derive from several overlapping mechanisms. Abnormal placentation involves altered trophoblast invasion and ECM remodeling, both of which are closely linked to fibronectin biology. Uteroplacental malperfusion, oxidative stress, and inflammation may modify protein glycosylation patterns and increase the release of altered ECM-related proteins into the maternal circulation. Endothelial dysfunction may also be associated with increased fibronectin expression, matrix remodeling, and vascular injury. Finally, because fibronectin participates in angiogenesis and cell-matrix signaling, altered GlyFn levels may reflect disruption of pathways that are essential for placental vascular development. These mechanisms provide a biological rationale for investigating GlyFn in placenta-mediated disorders, including FGR caused by placental insufficiency.

For this reason, GlyFn should be interpreted as a biologically plausible marker of placental and endothelial remodeling, with clinical utility in FGR still requiring validation.

Glycosylated fibronectin in pregnancy complications

The clinical investigation of GlyFn has expanded from its biological role as a modified ECM glycoprotein toward its potential use as a circulating biomarker of adverse pregnancy outcomes. This interest is based on the observation that several major pregnancy complications share overlapping biological pathways, including abnormal placentation, endothelial dysfunction, inflammation, metabolic disturbance, oxidative stress, altered angiogenesis, and ECM remodeling. Current evidence has focused mainly on preeclampsia, although GlyFn has also been evaluated in gestational diabetes mellitus (GDM), spontaneous preterm birth, and broader placenta-mediated complications, including fetal growth restriction [8-11,29-31].

Among pregnancy complications, preeclampsia is the condition in which GlyFn has been most extensively studied. Preeclampsia is a multisystem disorder characterized by new-onset hypertension after 20 weeks of gestation, with or without proteinuria, and may involve maternal organs, the uteroplacental circulation, and fetal growth. Its pathophysiology overlaps substantially with placenta-mediated FGR, particularly in early-onset disease, through abnormal trophoblast invasion, defective spiral artery remodeling, placental malperfusion, angiogenic imbalance, oxidative stress, and maternal endothelial dysfunction [16,19,20]. Because fibronectin and its glycosylated forms are linked to endothelial activation and ECM remodeling, GlyFn has been proposed as a candidate biomarker capable of reflecting these underlying biological processes.

Initial studies suggested that maternal serum GlyFn levels are elevated in women with preeclampsia and may help distinguish affected from unaffected pregnancies. Rasanen et al. evaluated maternal serum GlyFn as a point-of-care biomarker for the assessment of preeclampsia and reported its potential clinical utility in identifying women with disease [30]. Huhn et al. subsequently assessed GlyFn in a prospective cohort of women at increased risk or with clinical signs and symptoms of preeclampsia, comparing its performance with established biomarkers, including PlGF, sFlt-1, and pregnancy-associated plasma protein-A2 (PAPP-A2). In that study, GlyFn demonstrated strong short-term predictive performance for preeclampsia, supporting its role as a candidate biomarker for triage and risk assessment in symptomatic or high-risk pregnancies [8]. Point-of-care testing approaches have further increased interest in GlyFn because they may allow rapid assessment in clinical settings where timely differentiation between true disease and nonspecific symptoms is essential [9,30].

More recent evidence has strengthened the association between GlyFn and preeclampsia. A systematic review and meta-analysis including 11 studies found that maternal serum GlyFn levels were significantly higher in women with preeclampsia than in controls, with a standardized mean difference of 1.08 and an area under the summary receiver-operating characteristic curve of 0.90 for preeclampsia diagnosis [10]. The same meta-analysis reported pooled sensitivity and specificity of 0.81 and 0.80, respectively, although substantial heterogeneity was observed, partly related to differences in assay methods, study design, control populations, gestational age at sampling, and diagnostic thresholds [10]. These findings indicate that GlyFn has promising diagnostic performance in preeclampsia, but they also highlight the need for assay standardization and external validation before routine implementation.

GlyFn has also been evaluated in relation to preeclampsia-related complications. Kluivers et al. reported that GlyFn may be useful not only for preeclampsia diagnosis but also for identifying preeclampsia-related complications, particularly before 37 weeks of gestation [11]. This observation is relevant to FGR research because fetal growth restriction may occur as part of the same placenta-mediated disease spectrum that produces maternal hypertension and endothelial dysfunction. However, evidence from preeclampsia-related complications should be interpreted as indirect for FGR, because it does not establish GlyFn as an independent marker of isolated fetal growth restriction.

Beyond preeclampsia, GlyFn has been studied as a potential early biomarker for GDM. In a case-control study, Rasanen et al. measured first-trimester maternal serum concentrations of *Sambucus nigra* lectin-reactive GlyFn and other biomarkers in women who subsequently developed GDM and in controls. GlyFn was independently associated with later GDM after adjustment for maternal factors and other biomarkers, and it demonstrated stronger discriminatory performance than adiponectin, high-sensitivity C-reactive protein, and placental lactogen, with an area under the curve (AUC) of 0.91 [31]. At a threshold of 120 mg/L, GlyFn showed a high negative predictive value, suggesting potential utility for early identification of women at low risk of GDM [31]. These results supported the concept that altered fibronectin glycosylation may occur early in pregnancy and may be linked to metabolic as well as vascular pregnancy complications.

However, subsequent studies of GlyFn in GDM have produced inconsistent findings. A later first-trimester study found no statistically significant difference in GlyFn concentrations between women who developed GDM and controls, although differences in fibronectin concentrations and potential influences of body mass index and smoking were observed [32]. More recently, Lata et al. reported decreased first-trimester maternal serum GlyFn levels in women with GDM compared with non-GDM pregnancies and concluded that GlyFn showed only modest diagnostic accuracy for GDM, with an AUC of 72.4% [33]. These conflicting findings suggest that the relationship between GlyFn and GDM may depend on assay platform, glycoform specificity, gestational age at sampling, maternal metabolic phenotype, population characteristics, and study design. They also emphasize that GlyFn should not be interpreted as a uniformly increased marker across all pregnancy complications. Instead, changes in GlyFn may differ according to the dominant pathophysiological process and the specific glycosylation pattern measured.

Evidence linking GlyFn to spontaneous preterm birth is more limited but biologically plausible. Preterm birth is a syndrome with multiple causes, including infection, inflammation, decidual hemorrhage, cervical dysfunction, uterine overdistension, maternal disease, and placental pathology. Fibronectin biology is already clinically relevant in obstetrics through fetal fibronectin testing, which reflects disruption of the choriodecidual interface and is used in selected contexts to assess the risk of preterm delivery. GlyFn differs from fetal fibronectin, but both markers highlight the importance of ECM and cell-adhesion pathways in pregnancy maintenance. A review of GlyFn as a wide-spectrum biomarker identified spontaneous preterm birth as one of the pregnancy outcomes in which altered GlyFn or fibronectin glycosylation patterns may be relevant [29]. More recent clinical utility data have also suggested that positive or high-positive GlyFn results may be associated with preterm birth before 37 weeks, including among singleton pregnancies without preeclampsia, although these findings derive from a study primarily designed to assess impending preeclampsia [34]. These observations require cautious interpretation, but they support further investigation of GlyFn in preterm birth phenotypes, particularly those related to placental dysfunction or inflammatory activation.

The available evidence therefore supports GlyFn as a candidate biomarker across several pregnancy complications, but with important differences in the strength and consistency of the data. The most consistent evidence concerns preeclampsia, where several studies and meta-analytic findings support elevated maternal GlyFn levels and diagnostic potential [8-11,30]. Evidence in GDM is inconsistent, with studies reporting increased, unchanged, or decreased GlyFn levels depending on population and methodology [31-33]. Evidence in spontaneous preterm birth and other placenta-mediated complications remains preliminary [29,34]. Thus, GlyFn should be interpreted not as a disease-specific biomarker, but as a potential indicator of selected biological pathways that may operate across different obstetric syndromes.

This interpretation is relevant to fetal growth restriction because major obstetric syndromes often overlap clinically and biologically. Preeclampsia, FGR, placental abruption, spontaneous preterm birth, and stillbirth have been conceptualized as “great obstetrical syndromes” that may arise from disorders of deep placentation and maternal-fetal interface dysfunction [15]. GlyFn may fit within this framework because its biological associations include trophoblast-matrix interaction, endothelial activation, ECM remodeling, inflammation, and vascular stress. A pregnancy complicated by abnormal placentation may therefore show altered GlyFn before a specific clinical phenotype, such as hypertension or impaired fetal growth, becomes fully apparent. However, this concept remains hypothesis-generating.

Several limitations apply across the current GlyFn literature. First, most evidence remains concentrated in preeclampsia cohorts, and results cannot be automatically extrapolated to all placenta-mediated complications. Second, GlyFn does not necessarily refer to a single uniform molecular entity; different assays may detect different glycosylation patterns or lectin-reactive fibronectin fractions. Third, GlyFn levels may be influenced by maternal characteristics such as body mass index, smoking, inflammation, metabolic status, gestational age, and comorbid disease. Fourth, studies have used different sampling windows, clinical definitions, and diagnostic thresholds, making direct comparison difficult. Finally, many available studies are case-control or enriched high-risk cohorts rather than unselected prospective populations, which may overestimate diagnostic performance.

In relation to fetal growth restriction, these limitations are particularly important. Because FGR is heterogeneous, GlyFn may be most informative in phenotypes driven by placental malperfusion, endothelial dysfunction, and abnormal ECM remodeling. Thus, evidence from preeclampsia, GDM, preterm birth, and other placenta-mediated disorders provides a rationale for studying GlyFn in FGR, but direct validation in isolated and well-defined FGR cohorts remains necessary.

Glycosylated fibronectin and fetal growth restriction

GlyFn has attracted increasing interest as a circulating biomarker of placenta-mediated disease. However, its role in FGR must be interpreted separately from the substantially larger body of evidence concerning preeclampsia. Although several studies and meta-analytic data have demonstrated an association between elevated GlyFn concentrations and preeclampsia, direct evidence supporting its diagnostic or prognostic utility in isolated FGR remains very limited [8,10,11]. Most available observations relevant to fetal growth have been derived indirectly from cohorts of women with suspected or confirmed preeclampsia, studies examining composite adverse pregnancy outcomes, or broader reviews of GlyFn across several pregnancy complications [8,10,11,34,35]. GlyFn should therefore currently be regarded as a biologically plausible marker of placental dysfunction rather than as an independently validated biomarker of isolated FGR.

The biological rationale for investigating GlyFn in FGR is nevertheless compelling. Placenta-mediated FGR is characterized by impaired trophoblast invasion, incomplete spiral artery remodeling, maternal vascular malperfusion, uteroplacental hypoperfusion, oxidative stress, altered angiogenic signaling, endothelial activation and ECM remodeling. These pathological pathways overlap with those implicated in preeclampsia, which is the pregnancy disorder in which GlyFn has been studied most consistently [8,10,11]. Fibronectin contributes to cell adhesion, ECM organization, angiogenesis, endothelial function and tissue repair. Consequently, altered fibronectin glycosylation may reflect qualitative changes in the maternal-placental interface resulting from pathological placentation [8,34]. In the context of FGR, GlyFn would therefore be expected to reflect placental and endothelial stress rather than reduced fetal size itself.

This distinction is important because FGR is not synonymous with a fetus being small for gestational age. A fetus may be constitutionally small while remaining physiologically healthy, whereas pathological FGR denotes the failure to achieve the genetically determined growth potential because of an adverse intrauterine environment, most commonly placental insufficiency. Studies defining FGR exclusively according to estimated fetal weight or birth weight below the 10th percentile may therefore include constitutionally small fetuses without clinically relevant placental disease. Such misclassification could attenuate any true association between GlyFn and placental dysfunction. By contrast, definitions incorporating severe fetal smallness, impaired growth velocity, abnormal uterine, umbilical or cerebral Doppler findings, or adverse perinatal outcomes are more likely to identify the phenotype in which GlyFn could be biologically relevant [8]. Future GlyFn studies must therefore distinguish pathological FGR from constitutionally small fetal size.

Evidence Specifically Concerning Isolated FGR

At present, evidence evaluating GlyFn specifically in pregnancies with isolated FGR, in the absence of preeclampsia or another hypertensive disorder, is sparse. No sufficiently large body of prospective studies has established whether maternal GlyFn concentrations differ consistently between pregnancies with isolated FGR, constitutionally small-for-gestational-age fetuses and normally grown controls. Similarly, there is currently no robust evidence demonstrating that GlyFn can independently diagnose isolated early- or late-onset FGR, predict its progression, or improve the prediction of perinatal outcomes beyond established clinical, biophysical and angiogenic markers [8,10,11].

The available literature also does not adequately demonstrate that GlyFn elevation is attributable to fetal growth restriction itself rather than to coexisting maternal endothelial dysfunction. This limitation is particularly important because FGR and preeclampsia frequently coexist and share several pathological mechanisms. Unless pregnancies with isolated FGR are analysed separately from pregnancies complicated by both FGR and preeclampsia, an apparent association between GlyFn and FGR may primarily reflect the hypertensive disease component. Consequently, current evidence does not support the use of GlyFn as a stand-alone diagnostic or prognostic test for isolated FGR.

Robust evaluation would require prospective studies with clearly phenotyped and mutually exclusive groups, including normally grown controls, constitutionally small fetuses, isolated early-onset FGR, isolated late-onset FGR, FGR with preeclampsia and preeclampsia without FGR. Without this separation, it remains impossible to determine whether GlyFn identifies pathological fetal growth restriction independently or merely reflects the broader maternal endothelial and placental abnormalities associated with preeclampsia [8,10,11].

Indirect Evidence Derived From Preeclampsia Studies

The strongest evidence supporting a possible relationship between GlyFn and FGR is indirect and originates predominantly from studies of preeclampsia. Huhn et al. evaluated maternal serum GlyFn as a short-term predictor of preeclampsia in a prospective cohort that also incorporated a specific definition of intrauterine growth restriction [8]. In that study, intrauterine growth restriction was defined as estimated fetal weight below the 10th percentile in combination with pathological Doppler findings, including an abnormal cerebroplacental ratio or increased uterine artery pulsatility index, or as birth weight below the third percentile [8]. This definition was clinically relevant because it attempted to distinguish pathological growth restriction from fetal smallness alone. Nevertheless, preeclampsia prediction was the principal objective of the study, and the findings cannot be considered direct validation of GlyFn in isolated FGR.

Additional evidence has been provided by studies examining maternal and fetal complications among women with suspected or confirmed preeclampsia. Kluivers et al. assessed GlyFn after 20 weeks of gestation and reported that it could contribute to the identification of maternal and fetal preeclampsia-related complications, particularly before 37 weeks [11]. These findings are relevant to FGR because fetal growth restriction may represent a fetal manifestation of the same placental disease process that causes maternal hypertension and systemic endothelial dysfunction. However, they do not demonstrate that GlyFn independently predicts FGR among normotensive pregnancies or pregnancies without preeclampsia [11].

Similarly, the systematic review and meta-analysis by Liao et al. demonstrated higher GlyFn concentrations and favourable diagnostic performance in women with preeclampsia [10]. The included evidence, however, focused primarily on the diagnosis of preeclampsia rather than on FGR as an isolated outcome. The findings therefore reinforce the interpretation of GlyFn as a marker of placenta-mediated and endothelial pathology but do not establish it as a validated biomarker of isolated FGR [10]. Evidence from preeclampsia cohorts should consequently be presented as supportive biological and clinical context rather than as direct evidence of FGR-specific diagnostic accuracy.

The potential relationship between GlyFn and placenta-mediated disease may be explained through several complementary mechanisms. First, GlyFn may reflect abnormal placentation. Impaired trophoblast invasion and defective spiral artery remodeling alter the ECM of the decidua and placenta. Because fibronectin is involved in trophoblast adhesion and migration, altered glycosylation may reflect the disruption of placental matrix biology [8,10]. Second, GlyFn may reflect maternal endothelial activation. Placental malperfusion and oxidative stress promote the release of placental factors and extracellular vesicles into the maternal circulation, contributing to systemic endothelial dysfunction. Fibronectin participates in endothelial-matrix interactions and vascular remodeling, and altered circulating glycoforms may therefore accompany endothelial injury or activation [8,10,11]. Third, GlyFn may form part of a broader inflammatory and reparative response involving placental ischemia-reperfusion injury, oxidative stress, trophoblast damage and tissue remodeling [34,35].

These mechanisms may be particularly relevant to early-onset FGR, which is more frequently associated with severe placental vascular pathology, maternal vascular malperfusion, abnormal uterine and umbilical artery Doppler findings and coexisting hypertensive disease. If GlyFn reflects placental malperfusion and endothelial activation, stronger associations might be expected in early-onset than in late-onset FGR [8,11]. This remains a biological hypothesis rather than a clinically validated distinction. Late-onset FGR may be associated with subtler placental abnormalities, normal umbilical artery Doppler findings and less pronounced angiogenic or endothelial disruption. GlyFn may consequently have lower sensitivity in late-onset disease or may require interpretation alongside PlGF, the sFlt-1/PlGF ratio, uterine artery Doppler, cerebroplacental ratio and longitudinal fetal growth velocity [35].

Potential Diagnostic and Prognostic Applications

There is currently no robust evidence that GlyFn alone can diagnose FGR. Before clinical implementation, studies must demonstrate that it can distinguish pathological FGR from constitutionally small fetal size and that its performance is reproducible across gestational ages and clinical populations. Importantly, diagnostic analyses must adjust for preeclampsia and other hypertensive disorders to determine whether GlyFn contributes FGR-specific information independently of maternal endothelial disease [8,10,11].

The prognostic application of GlyFn may be more plausible than its use as a diagnostic marker, although the evidence remains preliminary. Once a fetus has been identified as small, the principal clinical challenge is to determine whether placental dysfunction is present and to predict deterioration. A clinically valuable biomarker could contribute to identifying pregnancies at increased risk of Doppler deterioration, fetal hypoxemia, medically indicated preterm delivery, stillbirth, neonatal intensive care admission or severe placental pathology. GlyFn might theoretically assist with this risk stratification because it reflects biological pathways involved in placental stress, ECM remodeling and endothelial activation [11,34]. Evidence from preeclampsia-related cohorts suggests that GlyFn may identify pregnancies at increased risk of maternal or fetal complications, particularly at preterm gestations [36]. Nevertheless, its independent prognostic value in isolated FGR has not been established.

Another important consideration is whether GlyFn provides incremental information beyond established biomarkers. PlGF and the sFlt-1/PlGF ratio have been extensively studied in preeclampsia and are increasingly evaluated in pregnancies with suspected FGR. These markers predominantly reflect the balance between proangiogenic and antiangiogenic signalling. GlyFn may represent a partly different biological axis involving ECM remodeling, endothelial activation and altered protein glycosylation [8,34]. Its potential role may therefore lie in a multimarker strategy rather than in replacing angiogenic biomarkers. A model incorporating maternal characteristics, fetal biometry, Doppler indices, angiogenic biomarkers and GlyFn could theoretically improve the identification of clinically significant placental insufficiency [34,35]. Preliminary studies have investigated combinations such as GlyFn with PlGF or GlyFn/PlGF ratios in high-risk pregnancies, but these approaches remain investigational and require validation using FGR-specific outcomes [35].

Interpretation is further complicated by assay variability. GlyFn is not necessarily a single uniform molecular entity, and different analytical platforms may detect different GlyFn fractions or lectin-reactive glycoforms. This may partly account for heterogeneity among studies of preeclampsia and other pregnancy complications [10]. Future FGR studies should clearly report the assay platform, analytical characteristics, measurement units, gestational age-specific reference ranges, proposed thresholds and intra- and inter-assay reproducibility. Without assay standardization, comparisons across studies and the establishment of clinically meaningful cutoffs will remain difficult.

Placental histopathology may provide an important means of establishing whether GlyFn is genuinely associated with FGR-related placental disease. If GlyFn reflects placental dysfunction, maternal concentrations should correlate with lesions such as maternal vascular malperfusion, decidual arteriopathy, placental infarction, accelerated villous maturation, distal villous hypoplasia, increased fibrin deposition and syncytial knotting. Demonstrating such associations would strengthen its interpretation as a marker of placental pathology rather than as a nonspecific circulating protein abnormality [8,34]. Future studies should therefore incorporate standardized placental pathological examination, particularly in severe and early-onset FGR.

Longitudinal measurement may also be more informative than assessment at a single time point. FGR evolves over time, and the trajectory of placental dysfunction differs between early- and late-onset disease. A single GlyFn measurement may be influenced by gestational age, maternal characteristics, concurrent preeclampsia and analytical variability. Serial measurements could determine whether increasing GlyFn concentrations precede reduced fetal growth velocity, Doppler deterioration, the development of maternal hypertensive disease or medically indicated preterm delivery [8,11,35]. Nevertheless, until such longitudinal studies are performed specifically in isolated FGR, GlyFn should be considered an investigational biomarker whose FGR-related utility is supported mainly by biological plausibility and indirect evidence from preeclampsia rather than by direct clinical validation.

The main clinical and evidence-synthesis studies evaluating GlyFn in pregnancy complications relevant to FGR are summarized in Table 1.

**Table 1:** Clinical and evidence-synthesis studies of GlyFn in pregnancy complications relevant to FGR. FGR, fetal growth restriction; GDM, gestational diabetes mellitus; GlyFn, glycosylated fibronectin; IUGR, intrauterine growth restriction; PAPP-A2, pregnancy-associated plasma protein-A2; PlGF, placental growth factor; sFlt-1, soluble fms-like tyrosine kinase-1.

Author/year	Population	Gestational age at sampling	Outcome	Main GlyFn finding	Relevance to FGR	Main limitation
Rasanen et al., 2013 [31]	Women who later developed GDM and controls	First trimester	Prediction of GDM	*Sambucus nigra* lectin-reactive GlyFn was independently associated with later GDM and showed high discriminatory performance, with an AUC of 0.91.	Suggests altered fibronectin glycosylation may occur early in pregnancy and may relate to metabolic/vascular pregnancy complications.	GDM is not primarily a placental FGR phenotype; findings may not extrapolate to FGR.
Rasanen et al., 2015 [30]	Women evaluated for suspected or established preeclampsia	At clinical assessment	Preeclampsia diagnosis	Maternal serum GlyFn was evaluated as a point-of-care biomarker and was associated with preeclampsia.	Supports GlyFn as a marker of placenta-mediated and endothelial dysfunction, pathways shared with placental FGR.	Focused on preeclampsia rather than isolated FGR.
Huhn et al., 2020 [8]	Prospective cohort of women at increased risk or with signs/symptoms of preeclampsia	At short-term clinical assessment in high-risk or symptomatic pregnancies	Short-term prediction of preeclampsia; included Doppler-defined IUGR criteria	GlyFn showed strong short-term predictive performance for preeclampsia compared with established biomarkers, including PlGF, sFlt-1, and PAPP-A2.	Relevant because the study included a pathological IUGR definition incorporating fetal size and Doppler abnormalities.	Primary outcome was preeclampsia, not isolated FGR.
Nagalla et al., 2020 [9]	Pregnant women assessed in a low-resource Southeast Asian setting	At point-of-care testing for suspected preeclampsia	Diagnosis of preeclampsia	GlyFn point-of-care testing supported diagnostic assessment of preeclampsia.	Demonstrates feasibility of rapid GlyFn testing in clinical obstetric settings.	Does not directly evaluate FGR-specific diagnostic or prognostic performance.
Alanen et al., 2020 [32]	Women assessed for first-trimester prediction of GDM	First trimester	GDM	No statistically significant difference in GlyFn concentrations between women who developed GDM and controls; fibronectin concentrations and maternal factors may have influenced findings.	Shows that GlyFn changes are not uniform across pregnancy complications.	Inconsistent with earlier GDM data; influenced by assay, population, BMI, and smoking.
Liao et al., 2025 [10]	Systematic review and meta-analysis of 11 studies	Varied across included studies	Diagnostic accuracy for preeclampsia	GlyFn levels were significantly higher in preeclamptic pregnancies; summary AUC was 0.90, with pooled sensitivity and specificity of 0.81 and 0.80.	Strengthens the evidence that GlyFn reflects placenta-mediated and endothelial pathology.	Focused on preeclampsia; substantial heterogeneity; not direct evidence for isolated FGR.
Kluivers et al., 2025 [11]	Women with suspected or confirmed preeclampsia	After 20 weeks of gestation	Preeclampsia and preeclampsia-related complications	GlyFn was evaluated as a biomarker for preeclampsia and related maternal/fetal complications, particularly before 37 weeks.	Relevant because FGR may occur as a fetal manifestation of preeclampsia-related placental disease.	Does not prove independent prediction of isolated FGR without hypertensive disease.
Lata et al., 2025 [33]	Women with and without later GDM	First trimester	Prediction of GDM	GlyFn levels were decreased in women with GDM compared with non-GDM pregnancies and showed modest diagnostic accuracy, with an AUC of 72.4%.	Supports the concept that GlyFn directionality may depend on pregnancy phenotype and assay context.	Modest performance; GDM findings do not directly inform FGR diagnosis.
Di Renzo et al., 2025 [34]	Women assessed with the Lumella GlyFn test for impending preeclampsia	At clinical assessment	Assessment of impending preeclampsia and related clinical outcomes	Positive or high-positive GlyFn results were associated with increased risk of impending preeclampsia; associations with preterm birth before 37 weeks were also reported in some subgroup analyses.	Supports the possibility that GlyFn may reflect clinically relevant placental disease severity.	Evidence remains preeclampsia-centered and indirect for isolated FGR.
Wirawan et al., 2025 [35]	High-risk pregnant women from four hospitals in West Java, Indonesia	20-28 weeks of gestation	Prediction of preeclampsia in high-risk pregnancies	GlyFn was higher and PlGF lower in women who developed preeclampsia; the GlyFn/PlGF ratio showed stronger predictive performance than either biomarker alone.	Relevant to FGR only indirectly, because combined GlyFn and angiogenic-marker models may capture complementary placental pathways.	Small cohort; outcome was preeclampsia prediction, not FGR-specific validation.

Comparison with established biomarkers

The evaluation of GlyFn in fetal growth restriction must be considered in the context of existing clinical, sonographic, and biochemical markers of placental dysfunction. At present, fetal biometry and Doppler velocimetry remain the foundation of FGR diagnosis and monitoring, while angiogenic biomarkers such as placental growth factor and the soluble fms-like tyrosine kinase-1/placental growth factor ratio have gained increasing clinical relevance, particularly in preeclampsia and placenta-mediated disease [1,2,7,36]. GlyFn differs from these established markers because it may reflect ECM remodeling, endothelial activation, altered cell-matrix interaction, and tissue injury rather than angiogenic imbalance alone. This distinction is important because FGR is biologically heterogeneous, and no single biomarker is likely to capture all relevant disease pathways.

Placental growth factor is one of the most extensively studied biomarkers of placental dysfunction. PlGF is a proangiogenic member of the vascular endothelial growth factor family and is produced predominantly by the placenta during pregnancy. Low maternal circulating PlGF concentrations are associated with impaired placental development, preeclampsia, FGR, and other adverse pregnancy outcomes [7,36,37]. In pregnancies with suspected FGR, low PlGF may help identify fetuses with underlying placental pathology and distinguish placental FGR from constitutional smallness [37]. This is particularly relevant because fetal size alone cannot reliably differentiate healthy small fetuses from fetuses exposed to uteroplacental insufficiency. Compared with GlyFn, PlGF has a stronger evidence base in placental disease and is more directly linked to angiogenic function. However, PlGF primarily reflects one biological axis, namely impaired proangiogenic signaling, whereas GlyFn may capture matrix-related and endothelial injury pathways that are not fully represented by PlGF.

The sFlt-1/PlGF ratio has become one of the most clinically useful biochemical tools for suspected preeclampsia and placenta-mediated complications. sFlt-1 is an antiangiogenic factor that binds and neutralizes VEGF and PlGF, thereby contributing to endothelial dysfunction. An elevated sFlt-1/PlGF ratio reflects antiangiogenic imbalance and has strong diagnostic and prognostic performance in preeclampsia [20,36]. In FGR, the sFlt-1/PlGF ratio may be particularly informative in early-onset placental disease, where maternal vascular malperfusion and angiogenic imbalance are more prominent. Recent evidence suggests that the sFlt-1/PlGF ratio may help distinguish pathological FGR from constitutional smallness, although it should be regarded as an adjunctive marker rather than a standalone diagnostic test [38]. In comparison, GlyFn may provide complementary information because it is not primarily an angiogenic marker. A combined interpretation of GlyFn and angiogenic markers could theoretically improve risk stratification by capturing both vascular growth-factor imbalance and ECM or endothelial remodeling.

Pregnancy-associated plasma protein-A is another established maternal serum marker relevant to fetal growth. PAPP-A is routinely measured in first-trimester aneuploidy screening and is involved in insulin-like growth factor bioavailability through cleavage of insulin-like growth factor (IGF)-binding proteins. Low first-trimester PAPP-A has been associated with placental dysfunction, FGR, preterm birth, hypertensive disorders of pregnancy, and pregnancy loss [39,40]. Its strength lies in early pregnancy risk prediction, often before clinical evidence of impaired fetal growth has emerged. However, its predictive performance as an isolated marker is limited, and many pregnancies with low PAPP-A do not develop FGR. GlyFn may differ from PAPP-A because it may reflect ongoing placental or endothelial stress later in pregnancy rather than only early placental development. Therefore, PAPP-A and GlyFn could potentially provide information at different time points: PAPP-A as an early risk marker and GlyFn as a dynamic marker of evolving placental dysfunction.

Other first-trimester maternal serum markers, including free β-human chorionic gonadotropin, alpha-fetoprotein, inhibin A, and placental protein 13, have also been investigated in relation to FGR and placenta-mediated complications. These markers may reflect placental mass, trophoblast differentiation, endocrine function, or abnormal placental development. However, their individual predictive value for FGR is generally modest, and their clinical utility is greatest when combined with maternal risk factors, uterine artery Doppler, and other biomarkers. GlyFn may fit into this broader multimarker approach, particularly if future studies demonstrate that it adds independent predictive value beyond currently available serum markers.

Doppler velocimetry remains indispensable because it provides functional information about uteroplacental resistance, fetoplacental circulation, and fetal cardiovascular adaptation. Uterine artery Doppler reflects maternal uteroplacental vascular resistance and is particularly useful in identifying impaired placentation. Umbilical artery Doppler evaluates downstream placental resistance and is central to surveillance of early-onset FGR. Middle cerebral artery Doppler and cerebroplacental ratio help identify fetal circulatory redistribution, especially in late-onset FGR. Ductus venosus Doppler and computerized cardiotocography are especially important in severe early-onset FGR, where delivery timing must balance fetal deterioration against prematurity-related morbidity [2,41]. The TRUFFLE (Trial of Randomized Umbilical and Fetal Flow in Europe) trial established the importance of structured monitoring using Doppler and computerized fetal heart rate assessment in very preterm FGR [41]. Compared with Doppler, GlyFn would not directly measure fetal hemodynamic adaptation, but it could potentially identify placental biological stress before advanced Doppler deterioration becomes apparent.

The key potential advantage of GlyFn is therefore not that it replaces the existing tools, but that it may represent a different biological domain. PlGF and the sFlt-1/PlGF ratio reflect angiogenic imbalance, PAPP-A reflects early placental and IGF-related function, Doppler indices reflect vascular resistance and fetal adaptation, and ultrasound biometry reflects fetal size and growth trajectory. GlyFn may reflect ECM remodeling, endothelial activation, altered protein glycosylation, and placental tissue stress. If validated, it could become part of a layered assessment model in which clinical risk factors, fetal growth velocity, Doppler parameters, angiogenic biomarkers, and matrix-related biomarkers are interpreted together.

However, compared with established biomarkers, GlyFn currently has a weaker evidence base in FGR. PlGF and sFlt-1/PlGF have been studied across large prospective cohorts and are incorporated into clinical pathways for suspected preeclampsia in several settings, whereas GlyFn remains investigational and has been evaluated mainly in preeclampsia cohorts [8-11,36]. Direct evidence for isolated FGR is insufficient. Thus, GlyFn should presently be considered a promising candidate biomarker rather than a competitor to established angiogenic markers or Doppler surveillance. Its future value will depend on whether it improves diagnostic discrimination, risk prediction, or clinical decision-making when added to existing models.

Potential clinical applications and research priorities

The potential clinical applications of GlyFn in FGR can be conceptualized across several stages of care, including early risk assessment, differentiation of pathological FGR from constitutional smallness, risk stratification after suspected FGR has been identified, monitoring of high-risk pregnancies, prediction of adverse perinatal outcomes, and phenotyping for future clinical research. At present, these applications remain hypothetical and investigational, because GlyFn has not yet been validated as an independent diagnostic or prognostic biomarker for isolated FGR. Nevertheless, they provide a useful framework for future studies.

One possible application is the early identification of pregnancies at risk of placenta-mediated FGR. Because abnormal placentation begins in the first trimester, an ideal biomarker would detect impaired placental development before fetal growth restriction becomes sonographically evident. Current early prediction models use maternal characteristics, obstetric history, mean arterial pressure, uterine artery Doppler, PAPP-A, PlGF, and other placental markers. GlyFn could potentially contribute to such models if altered fibronectin glycosylation is shown to occur early in pregnancies that later develop placental FGR. Evidence from preeclampsia and gestational diabetes studies suggests that GlyFn changes may be detectable before clinical disease is fully established, but FGR-specific prospective data are still needed [8,31,32].

A second clinically important application is differentiation between constitutionally small fetuses and pathologically growth-restricted fetuses. This distinction remains one of the central challenges in fetal medicine. A fetus with an EFW below the 10th percentile may be healthy and constitutionally small, whereas another fetus with the same EFW may have placental insufficiency and increased risk of hypoxemia, stillbirth, and neonatal morbidity. A biomarker of placental dysfunction could help refine this distinction. Low PlGF and an elevated sFlt-1/PlGF ratio already show promise in identifying small fetuses with placental pathology [37,38]. GlyFn could theoretically add value if it identifies ECM remodeling or endothelial activation in small fetuses whose angiogenic profile is not clearly abnormal. However, this application would require prospective studies comparing GlyFn levels across normal-growth controls, constitutionally small fetuses, isolated FGR, and FGR associated with preeclampsia.

A third potential use is risk stratification once FGR has been suspected or diagnosed. In clinical practice, the diagnosis of FGR is only the first step. The more difficult question is which pregnancies are at highest risk of deterioration and therefore require intensified surveillance or earlier delivery. GlyFn could become clinically useful if future studies demonstrate that elevated or rising levels correlate with progression to abnormal Doppler findings, maternal hypertensive disease, fetal hypoxemia, preterm delivery, neonatal intensive care admission, or adverse neonatal outcome. This prognostic role may be more realistic than a purely diagnostic role, because GlyFn may reflect the biological severity of placental stress rather than fetal size itself.

GlyFn may also be investigated as a monitoring tool in high-risk pregnancies. Women with chronic hypertension, previous FGR, previous preeclampsia, autoimmune disease, renal disease, diabetes, thrombophilia, or abnormal uterine artery Doppler are at increased risk of placenta-mediated complications. Serial GlyFn measurement in such populations could theoretically identify pregnancies in which placental stress is increasing before overt fetal growth failure or Doppler deterioration. This approach would be particularly useful if GlyFn trajectories prove more informative than single measurements. For example, a rising GlyFn level across the second or third trimester might indicate progressive endothelial or placental matrix injury, whereas stable low levels might support less intensive surveillance in otherwise reassuring pregnancies. However, this remains speculative and requires longitudinal validation.

Another possible application is the prediction of adverse perinatal outcomes. A clinically useful FGR biomarker should not merely correlate with fetal size but should predict outcomes that matter, including stillbirth, fetal acidosis, emergency delivery for fetal compromise, neonatal intensive care admission, respiratory morbidity, hypoglycemia, necrotizing enterocolitis, sepsis, neurodevelopmental impairment, or composite severe neonatal morbidity. Existing GlyFn data from preeclampsia-related cohorts suggest potential associations with adverse outcomes, but this has not been established specifically for isolated FGR [11]. Future studies should therefore evaluate outcome-based endpoints rather than relying only on birthweight percentiles.

GlyFn should also be explored as part of a multimodal prediction model rather than as a standalone test. In modern obstetrics, no single parameter is sufficient to guide management in complex FGR. Clinical decisions depend on gestational age, fetal growth velocity, Doppler findings, maternal disease, amniotic fluid volume, fetal movements, cardiotocography, and neonatal resources. GlyFn may be most useful if integrated into algorithms with PlGF, the sFlt-1/PlGF ratio, uterine artery Doppler, umbilical artery Doppler, cerebroplacental ratio, and maternal clinical factors. Such multimarker models could help identify different biological phenotypes of FGR, including predominantly angiogenic, inflammatory, matrix-remodeling, or vascular-malperfusion patterns.

Future research should therefore begin with well-designed prospective cohort studies in clearly defined FGR populations. These studies should distinguish early-onset FGR from late-onset FGR, isolated FGR from FGR with preeclampsia, and pathological FGR from constitutional smallness. The use of consensus diagnostic criteria, including fetal size, growth velocity, Doppler abnormalities, and gestational age at onset, would improve comparability across studies [3]. Enrolling separate control groups of appropriately grown fetuses and constitutionally small fetuses would be particularly important to determine whether GlyFn reflects true placental disease.

Longitudinal measurement of GlyFn should be prioritized. Serial sampling across gestation would clarify whether GlyFn changes precede the clinical diagnosis of FGR, correlate with disease progression, or predict deterioration. Longitudinal trajectories may be more informative than single thresholds, particularly in high-risk pregnancies. Future studies should evaluate whether rising GlyFn levels are associated with worsening umbilical artery Doppler, reduced cerebroplacental ratio, abnormal ductus venosus Doppler, maternal hypertensive disease, medically indicated preterm birth, or neonatal morbidity.

Combination biomarker panels represent another important research direction. GlyFn should be evaluated alongside PlGF, the sFlt-1/PlGF ratio, PAPP-A, inflammatory markers, oxidative stress markers, placental extracellular vesicles, cell-free placental RNA, and metabolomic or proteomic signatures. Because FGR is multifactorial, multimarker models are more likely to perform well than single biomarkers. GlyFn may be particularly useful if it contributes independent information related to ECM remodeling and endothelial activation. Recent work on combined angiogenic biomarkers and GlyFn/PlGF-based approaches suggests that multimarker strategies are feasible, but FGR-specific validation is still required [35].

Integration with Doppler and placental imaging should also be pursued. Biomarker data should not be interpreted in isolation but in relation to uterine artery Doppler, umbilical artery Doppler, middle cerebral artery Doppler, cerebroplacental ratio, ductus venosus Doppler, fetal growth velocity, placental volume, placental vascularization indices, and magnetic resonance imaging of placental structure and perfusion where available. Such integration could help determine whether GlyFn corresponds more closely to maternal vascular malperfusion, fetal circulatory adaptation, or generalized placental stress.

Placental histopathology will be essential for biological validation. Future studies should include standardized placental examination using accepted criteria such as the Amsterdam classification [17]. Correlating maternal GlyFn levels with lesions of maternal vascular malperfusion, decidual arteriopathy, infarction, distal villous hypoplasia, accelerated villous maturation, intervillous fibrin deposition, and syncytial knotting would clarify whether GlyFn is truly a marker of placental pathology. It would also help determine whether GlyFn is associated with specific histological phenotypes rather than FGR as a broad clinical category.

Finally, GlyFn may help define biologically meaningful phenotypes for future research and clinical trials. FGR is a heterogeneous syndrome, and trials of preventive or therapeutic strategies may fail if they include biologically diverse populations. GlyFn could potentially identify a subgroup of FGR characterized by ECM remodeling and endothelial activation. Such phenotyping may be valuable for future trials of therapies targeting placental vascular function, inflammation, oxidative stress, or endothelial pathways. However, this remains speculative until GlyFn is linked to reproducible placental histopathological patterns and clinically meaningful outcomes.

Limitations and requirements for clinical interpretation and application

Despite its biological plausibility, the current evidence supporting GlyFn in FGR has important limitations. The most significant limitation is that most GlyFn research has focused on preeclampsia rather than isolated FGR. Preeclampsia and FGR share several pathophysiological mechanisms, but they are not identical clinical entities. A biomarker that performs well in preeclampsia may reflect maternal endothelial dysfunction, hypertension-related vascular injury, or systemic inflammation rather than fetal growth restriction itself. Therefore, evidence from preeclampsia cohorts can support biological plausibility but cannot establish GlyFn as an FGR-specific biomarker.

A second limitation is phenotypic heterogeneity. FGR includes early- and late-onset disease, severe and mild forms, cases with and without hypertensive disorders, cases with abnormal or normal umbilical artery Doppler, and cases caused by placental, fetal, infectious, genetic, or maternal environmental factors. If these phenotypes are analyzed together, biomarker associations may be diluted or misleading. GlyFn may be more strongly associated with early-onset placental FGR than with late-onset or non-placental FGR. Without careful classification, it is impossible to determine its true clinical value.

A third limitation is the inconsistent definition of FGR and related outcomes. Some studies use estimated fetal weight below the 10th percentile, others use birthweight below the 10th percentile, while others use severe smallness below the third percentile, Doppler abnormalities, or composite adverse outcomes. These definitions identify overlapping but not identical populations. Studies using only birthweight or fetal size thresholds may include constitutionally small fetuses, whereas studies incorporating Doppler and placental pathology are more likely to identify true placental insufficiency. This definitional inconsistency is especially problematic for GlyFn because its proposed biological relevance is linked to placental dysfunction rather than smallness alone.

Assay heterogeneity is another major issue. GlyFn is not a single uniform analyte in the same way that PlGF or sFlt-1 are typically conceptualized. Different assays may detect different glycosylated forms of fibronectin or different lectin-reactive fractions. Variability in assay platform, antibody specificity, calibration, units, detection limits, and sample handling may contribute to differences between studies. The recent meta-analysis of GlyFn in preeclampsia identified significant heterogeneity, emphasizing the need for standardization before clinical translation [10]. This issue will be equally important in FGR research.

Gestational age at sampling is also critical. Biomarker concentrations may vary across pregnancy, and the timing of sampling relative to disease onset may influence diagnostic performance. A GlyFn value measured in the first trimester may reflect early placental development, whereas a value measured in the third trimester may reflect established placental stress, endothelial activation, or impending clinical deterioration. Studies that combine broad gestational age windows without appropriate adjustment may produce unreliable thresholds. FGR research should therefore establish gestational age-specific reference ranges and evaluate longitudinal trajectories.

Another limitation is the lack of robust comparison with established tools. For GlyFn to become clinically useful, it must add information beyond fetal biometry, Doppler indices, PlGF, the sFlt-1/PlGF ratio, PAPP-A, and maternal clinical risk factors. Many biomarker studies demonstrate statistical associations but do not show incremental predictive value, improvement in discrimination, net reclassification, or clinical utility. Future studies should assess whether GlyFn improves prediction models using measures such as change in AUC, calibration, decision-curve analysis, and outcome-based risk stratification.

Outcome-based validation is particularly important. Future research should move beyond the diagnosis of FGR and evaluate whether GlyFn predicts clinically meaningful outcomes, including stillbirth, fetal acidosis, emergency cesarean delivery for fetal compromise, neonatal intensive care admission, respiratory morbidity, hypoglycemia, severe neonatal morbidity, and long-term neurodevelopment. A biomarker that identifies small fetuses but does not improve prediction of adverse outcomes would have limited clinical value. Conversely, a biomarker that identifies high-risk fetuses among those with suspected FGR could meaningfully improve care.

There is also limited evidence that GlyFn-guided management improves clinical outcomes. Even if GlyFn predicts FGR or adverse outcomes, clinical implementation would require evidence that using the test changes management in a way that improves maternal or neonatal outcomes. Earlier detection may be beneficial if it leads to appropriate surveillance, corticosteroid administration, magnesium sulfate use, referral to tertiary care, or optimized delivery timing. However, unnecessary intervention could increase iatrogenic prematurity. Therefore, clinical utility studies are essential before GlyFn can be recommended for routine FGR management.

Confounding factors must also be considered. GlyFn levels may be influenced by maternal body mass index, smoking, diabetes, inflammation, renal disease, autoimmune disease, gestational age, preeclampsia, and other comorbidities. These factors are common in high-risk obstetric populations and may independently alter endothelial function, glycosylation pathways, or fibronectin metabolism. Studies must adjust for these variables and determine whether GlyFn remains independently associated with FGR-specific outcomes.

Finally, assay standardization is a prerequisite for clinical translation. Future studies should clearly define the GlyFn isoform or glycosylation pattern measured, report analytical performance, establish gestational age-specific reference ranges, and determine reproducible thresholds. Without such standardization, results cannot be reliably compared across cohorts or translated into clinical practice.

## Conclusions

GlyFn is an emerging biomarker with strong biological relevance to placenta-mediated pregnancy complications. Its connection to extracellular matrix remodeling, cell adhesion, trophoblast-matrix interaction, angiogenesis, endothelial activation, inflammation, and tissue injury provides a plausible mechanistic basis for its investigation in fetal growth restriction. Since pathological FGR is frequently driven by abnormal placentation, uteroplacental malperfusion, oxidative stress, altered angiogenic balance, and endothelial dysfunction, GlyFn may reflect important aspects of the disease process that are not fully captured by fetal biometry or Doppler assessment alone. However, the current evidence does not yet support GlyFn as an established diagnostic or prognostic biomarker for isolated FGR. Most available studies have focused on preeclampsia or preeclampsia-related complications, and direct evidence in well-defined FGR cohorts remains limited. Therefore, GlyFn should currently be regarded as a promising investigational marker of placental dysfunction rather than a clinically validated test for fetal growth restriction. Its most realistic future role may be as part of a multimodal prediction model. In combination with maternal risk factors, fetal growth velocity, Doppler velocimetry, PlGF, sFlt-1/PlGF ratio, PAPP-A, and placental imaging, GlyFn may contribute complementary information related to extracellular matrix and endothelial pathways. To establish this role, future studies must use standardized FGR definitions, separate early- and late-onset disease, distinguish isolated FGR from preeclampsia-associated FGR, include constitutionally small comparison groups, standardize GlyFn assays, incorporate placental histopathology, and evaluate clinically meaningful perinatal outcomes. Until such evidence is available, GlyFn should be considered a complementary research biomarker with translational potential rather than a standalone clinical tool for the diagnosis or management of fetal growth restriction.
